# Haemorrhagic transformation following ischaemic stroke: A retrospective study

**DOI:** 10.1038/s41598-020-62230-5

**Published:** 2020-03-24

**Authors:** S. D. Pande, M. M. Win, A. A. Khine, E. M. Zaw, N. Manoharraj, L. Lolong, A. S. Tin

**Affiliations:** 1 Department of Rehabilitation medicine, Changi General Hospital, Singapore, Singapore; 2 Clinical Trials and Research Unit, Changi General Hospital, Singapore, Singapore

**Keywords:** Stroke, Stroke

## Abstract

The aim of this study was to identify the prevalence of haemorrhagic transformation (HT) in patients with ischaemic stroke, and evaluate its association with medical comorbidities, stroke subtypes, premorbid medication, and long-term survival. To achieve this, we performed a retrospective analysis of 527 consecutive stroke rehabilitation patients. Of these, 102 (19.4%) developed HT. Older patients, and those with large artery strokes, had a higher risk of HT. Forty-one patients received alteplase (rtPA), of which 15 (36.6%) developed HT. A total of 129 (24.5%) patients were taking aspirin prior to their stroke and, of these, 39 (30.2%) developed HT. Twenty-three (4.36%) patients were taking vitamin k antagonists, prior to stroke, of which 14 (60.9%) developed HT. There were 102 patients (19.35%) with underlying atrial fibrillation, of whom 55 (53.9%) developed HT. Patients with known ischaemic heart disease had an increased risk of HT, and patients with HT had significantly lower total cholesterol levels (4.96 vs. 5.34) and lower LDL cholesterol levels (3.20 vs. 3.5). In conclusion, older age, atrial fibrillation, treatment with oral anticoagulants and antiplatelet medications prior to stroke, low total and LDL cholesterol, and rtPA use, are all associated with HT. Survival was not affected by the presence of HT.

## Introduction

Haemorrhagic transformation (HT) is a complication of ischemic stroke, and was described in the 19^th^ century by Cohnheim (1872) and Liddel (1873). HT has been described as ‘cerebral red softening identified as presence of netlike haemorrhage in acutely degenerating grey matter’. HT is a term used synonymously to describe haemorrhagic infarction that occurs following venous thrombosis or arterial thrombosis and embolism^1^.

HT has been shown to occur at different time points following ischaemic stroke, and may or may not present with a neurological deterioration. Early HT occurs as a result of the recirculation of blood due to clot movement, leading to reperfusion via leptomeningeal anastomoses^2^. Late HT is thought to be due to increased vascular permeability and increased blood flow, following a reduction in cerebral oedema^3^. HT is found more commonly in autopsy studies^4^. With the increasing use of rtPA in acute stroke, various studies have reported an increase in the incidence of HT, and improved imaging techniques mean that HT is being diagnosed with higher accuracy. Since the early descriptions of HT, various studies have suggested that haemorrhagic transformation is probably the natural progression of ischemic stroke^3,5–7^. HT may result in neurological deterioration^7–9^, and the presence of a parenchymal hematoma (PH) has been shown to lead to a significant clinical deterioration^7,9–16^.

Hornig *et al*. found that 43% of 65 patients with ischaemic stroke had HT, which was associated with mass effect and severe neurological deficits, and the risk of HT was higher in patients with large infarctions^3^. Higher National Institutes of Health Stroke Scale (NIHSS) score, proximal MCA occlusion, hypodensity of the middle cerebral artery territory, and delayed recanalization (6 hours or more after stroke onset) have all been shown to be associated with HT^17,18^.

As a significant number of patients with ischaemic stroke are transferred for inpatient rehabilitation, HT may be detected during this period. HT may also affect the decision to restart antiplatelets or anticoagulants, and thus the factors which contribute to HT are important. This particular group of patients should be monitored carefully for neurological deterioration, and undergo serial brain scans.

The aim of our study was to investigate the incidence and underlying factors contributing to HT, and its effect on long-term survival following rehabilitation as compared to ischemic stroke patients that did not experience HT.

## Methods

### Study population, inclusion and exclusion criteria

The SingHealth Centralized Institutional Review Board (IRB) approved this study (approval number: 2015/3112) and the need for informed consent was waived due to the retrospective nature of the study. Data collection was censored in May 2017. All collected material was stored according to the SingHealth IRB policy in the hospital’s medical record database, and the clinician records were stored at the neurorehabilitation facility.

Changi General Hospital is a government-restructured hospital with modern facilities for emergency and specialist care. The Acute Stroke Unit is equipped with facilities for diagnosis and treatment, including thrombolysis. All patients admitted with a diagnosis of stroke undergo the necessary investigations, including the tests required to establish the underlying cause of the stroke, according to standardized protocols and established guidelines. Stroke management is streamlined, and involves the Emergency Department and Acute Stroke Unit. On the basis of the initial and subsequent clinical status and the scan findings, the neurosurgery team is involved for further interventions, where required. All patients are eventually referred to the inpatient neurorehabilitation department, and are regularly followed-up after discharge.

For the present study, we reviewed all electronic and paper medical records (covering initial and subsequent admissions, as well as follow-up visits, and including all administered treatments) of consecutive patients aged 21 years or older with stroke (including infarction and haemorrhagic transformation), who were admitted to the neurorehabilitation facility at Changi General Hospital, Singapore between June 2008 and May 2017. For the survival analysis, the minimum follow-up period was six months.

Patients were excluded from this study if they were aged below 21 years and/or had an initial diagnosis of primary spontaneous intracerebral haemorrhage (SICH) or traumatic intracerebral haemorrhage.

### Evaluation of stroke

The subtype, severity, and location of stroke were diagnosed on admission to the Acute Stroke Unit by a stroke physician or neurosurgeon, who performed clinical examinations along with brain imaging, including computed tomography (CT), magnetic resonance imaging (MRI), and magnetic resonance angiography (MRA). Patients were categorized according to the presence of ischaemic stroke and haemorrhagic stroke on the basis of the imaging findings. Records of patients receiving thrombolysis treatment were maintained, along with the findings of repeat scans for suspected neurological deterioration and haemorrhagic conversion.

Patients developing HT within 48 hours were labelled as early and those detected beyond 48 hours were labelled as late HT^19^.

Cardioembolic stroke was diagnosed using 12-lead Holter electrocardiography, carotid Doppler imaging, and echocardiography. Other haematological, biochemistry, and autoimmune tests were conducted to rule out secondary causes of stroke.

The location of the stroke was classified using the Oxfordshire system^20^, as follows: total anterior circulation stroke (TACS), partial anterior circulation stroke (PACS), lacunar stroke (LACS), and posterior circulation stroke (POCS).

The aetiology of stroke was classified using the Trial of Org 10172 in Acute Stroke Treatment (TOAST) system^21^: large-artery atherosclerosis (LAA), small vessel occlusion (SVO), cardioembolism (CE), stroke of other determined aetiology, and stroke of undetermined aetiology. The probability of a cardioembolic source was also assessed as moderate or high.

### Evaluation of stroke treatments

Treatments, including thrombolysis with alteplase (rtPA), antiplatelets, anticoagulants, and neurosurgical procedures (including burr hole drainage, external ventricular drainage [EVD], intracranial pressure [ICP] monitoring, craniotomy, and craniectomy), were all documented.

Preadmission medications, including platelet inhibitors, aspirin, clopidogrel other vitamin k antagonists, newer oral anticoagulants, and dipyridamole, were all documented.

Regarding the diagnosis of haemorrhagic transformation, all admission and follow-up scans were reported by radiologists. Neurology or neurosurgery reviews were obtained regarding the diagnosis of HT, the need to repeat brain scans, the timing of restarting antiplatelets or anticoagulants, and possible neurosurgical interventions.

### Summary of evaluated parameters

The demographic details, diagnosis, stroke type and location, CT/MRI findings, electrolyte levels, clotting parameters, premorbid medications, and comorbidities (hypertension, diabetes, hyperlipidaemia, atrial fibrillation [AF]) were recorded at the time of admission for all patients. Treatment data were also documented, and included the use of thrombolysis with rtPA, medical treatments for increased intracranial pressure (mannitol), and neurosurgical interventions.

### Statistical analysis

Categorical data are presented as frequency (percentage) and continuous data are presented as mean (± standard deviation) for parametric distributions, or median (interquartile range) for nonparametric distributions. Differences between subgroups were examined using Chi-square tests for categorical variables and two-sample t-tests for continuous variables. Odds ratios (ORs) were presented along with 95% confidence intervals (CIs). A two-tailed p-value of <0.05 was considered statistically significant. All statistical analyses were performed using Statistical Package for the Social Sciences (SPSS) version 19.0 (IBM Corp. Armonk, New York, USA).

## Results

In total, 713 patient records were reviewed, and of these, 186 had a primary intracerebral haemorrhage, and 3 had traumatic intracerebral haemorrhage. Of the remaining 527 patients who were diagnosed with ischaemic stroke on admission, 102 (19.4%) experienced HT. Of these 102, 88 (86.3%) were classified as ‘early HT’ (within 48 hours), and 14 (13.7%) were classified as ‘late HT’ (beyond 48 hours). Of all those experiencing HT, 17 (16.7%) required treatment to lower ICP, and 7 (6.9%) needed neurosurgical intervention. Those with large artery ischaemic strokes had a higher risk of HT [(94) 25.2% vs (8) 5.2%, p = 0.001) than those with small artery strokes.

Of the 527 patients with ischaemic stroke, 102 (19.4%) had underlying AF, and 55 (53.9%) of these patients developed HT (p = 0.001). Of all the subjects, 23 (4.4%) were taking vitamin k antagonists, and fourteen (60.9%) of these developed HT (p = 0.001). Logistic regression analysis indicated that AF remained a significant and independent risk factor for HT in those on vitamin k antagonists.

Forty-one (7.8%) of the 527 patients with ischaemic stroke received rtPA, of which 15 (36.6%) developed HT (p = 0.004). Those who received rtPA had a higher risk of HT (Table 1). Patients with large artery strokes and a high risk of cardioembolism were also significantly more likely to have haemorrhagic transformation. Using the stroke territory classification, patients with TACS (35.7%) and PACS (35.6%) had a higher risk of HT.

**Table 1:** Characteristics and classification of haemorrhagic transformation.

Characteristics of patients who had [Haemorrhage transformation = HT] events (n = 102)		
age, mean (SD), [years]		68.4 (11.5)
Gender, no (%)	male	60 (58.8)
	female	42 (41.2)
Ethnicity, no (%)	Chinese	62 (60.8)
	Indian	11 (10.8)
	Malay	27 (26.5)
	Others	2 (2.0)
Duration of HT, no (%)	Early	88 (86.3)
	Late	14 (13.7)
Small petechiae, no (%)	No	67 (65.7)
	Yes	35 (34.3)
More confluent petechiae, no (%)	No	68 (66.7)
	Yes	34 (33.3)
PH 1, no (%)	No	82 (80.4)
	Yes	20 (19.6)
PH2, no (%)	No	89 (87.3)
	Yes	13 (12.7)
SAH, no (%)	No	102 (100)
	Yes	0 (0)
IVH, no (%)	No	101 (99)
	Yes	1 (1)
SDH, no (%)	No	102 (100)
	Yes	0 (0)
Surgical intervention, no (%)	No	95 (93.1)
	Yes	7 (6.9)
Medical treatment for increased ICP, no (%)	No	85 (83.3)
	Yes	17 (16.7)
Indication of repeat scan, no (%). N = neurological deterioration. S = stroke protocol. T = thrombolysis. O = other indication.	N	35 (34.3)
	S	56 (54.9)
	T	10 (9.8)
	O	1 (1.0)
Stroke territory, no (%)	TACS	10 (9.8)
	PACS	61 (59.8)
	LACS	11 (10.8)
	POCS	20 (19.6)

Abbreviation: PH: parenchymal haematoma (type 1 = less than 30%, type 2 = more than 30%). SAH = subarachnoid haemorrhage,

IVH = intraventricular haemorrhage, TACS = total anterior circulation syndrome, PACS = partial anterior circulation syndrome, LACS = lacunar syndrome, POCS = posterior circulation syndrome.

There were 129 (24.5%) patients taking aspirin and, of these, 39 (30.2%) developed HT (p = 0.001). Those on dipyridamole, clopidogrel, statins, or fibrate did not appear to have a significantly increased risk of HT (Table 2). Patients with HT had a significantly lower total cholesterol (4.96 vs 5.34, p = 0.018) and lower LDL cholesterol (3.20 vs 3.5, p = 0.023; Table 3). Old age was significantly associated with the risk of HT (68.4 vs 65.4, p = 0.001), as were higher potassium levels (4.25 vs 4.07, p = 0.007). Altered kidney function, full blood count and liver function tests did not show any relationship with HT.

**Table 2:** Haemorrhagic transformation in relation to premorbid medications,.

		Haemorrhagic transformation.		Total	p-value	test
		No	Yes			
		425 (81%)	102 (19.4%)	527		
Age, mean (SD)		65.4 (12.4)	68.4 (11.5)		0.027	t-test
Gender n, (%)	male	258, (81.1)	60, (18.9)	318	0.727	chi-square
	female	167, (80)	42, (20)	209		
Race, n, (%)	Chinese	253, (80.3)	62, (19.7)	315	0.445	chi-square
	Indian	41, (78.8)	11, (21.2)	52		
	Malay	106, (79.7)	27, (20.3)	133		
	Others	25, (92.6)	2, (7.4)	27		
Use of NSAID, n, (%)	No	412, (80.5)	100, (19.5)	512	0.549	chi-square
	Yes	13, (86.7)	2, (13.3)	15		
Use of Aspirin, n, (%)	No	335, (84.2)	63, (15.8)	398	<0.001	chi-square
	Yes	90, (69.8)	39, (30.2)	129		
Use of clopidogrel, n, (%)	No	399, (80.9)	94, (19.1)	493	0.524	chi-square
	Yes	26, (76.5)	8, (23.5)	34		
Use of statin, n, (%)	No	265, (82.6)	56, (17.4)	321	0.166	chi-square
	Yes	160, (77.7)	46, (22.3)	206		
Use of fibrate, n, (%)	No	403, (80.6)	97, (19.4)	500	0.91	chi-square
	Yes	22, (81.5)	5, (18.5)	27		
Use of warfarin, n, (%)	No	416, (82.5)	88, (17.5)	504	<0.001	chi-square
	Yes	9, (39.1)	14, (60.9)	23		
Use of dipyridamole, n, (%)	No	416, (80.9)	98, (19.1)	514	0.292	chi-square
	Yes	69.2	30.8	13		
Hypertension, n, (%)	No	125, (83.3)	25, (16.7)	150	0.324	chi-square
	Yes	300, (79.6)	77, (20.4)	377		
Diabetes (%)	No	233, (81.5)	53, (18.5)	286	0.602	chi-square
	Yes	192, (79.7)	49, (20.3)	241		
Lipids (%)	No	200, (82)	44, (18)	244	0.476	chi-square
	Yes	225, (79.5)	58, (20.5)	283		
IHD (%)	No	338, (84.9)	15.1	398	<0.001	chi-square
	Yes	87, (67.4)	42, (32.6)	129		
AMI (%)	No	415, (80.3)	102, (19.7)	517	0.118	chi-square
	Yes	10, (100)	0, (0)	10		
Angioplasty (%)	No	415, (80.4)	101, (19.6)	516	0.448	chi-square
	Yes	9, (90)	1, (10)	10		
AF (%)	No	378, (88.9)	47, (11.1)	425	<0.001	chi-square
	Yes	47, (46.1)	55, (53.9)	102		
Kidney function (%)	Normal & AKI	326, (80.9)	77, (19.1)	403	0.795	chi-square
	CKD	99, (79.8)	25, (20.2)	124		
Total Cholesterol, mean (SD)		5.34 (1.46)	4.96 (1.55)		0.018	t-test
HDL, mean (SD)		1.17 (0.43)	1.17 (0.55)		0.964	t-test
Total HDL ratio, mean (SD)		4.9 (1.7)	4.7 (2.1)		0.448	t-test
Triglyceride, mean (SD)		1.49 (0.86)	1.36 (0.09)		0.151	t-test
LDL, mean (SD)		3.5 (1.28)	3.20 (1.46)		0.023	t-test
Urea, mean (SD)		5.49 (3.5)	5.33 (2.29)		0.664	t-test
Sodium, mean (SD)		137.45 (3.6)	137.57 (3.89)		0.754	t-test
Potassium, mean (SD)		4.07 (0.58)	4.25 (0.75)		0.007	t-test
Glucose, mean (SD)		10.09 (5.45)	10.9 (6.9)		0.205	t-test
Creatinine, mean (SD)		103.98 (81.0)	96.7 (39.8)		0.375	t-test
Hb, mean (SD)		14.1 (4.8)	14.7 (2.0)		0.909	t-test
WBC, mean (SD)		9.6 (3.2)	9.9 (3.9)		0.388	t-test
Platelet, mean (SD)		281.7 (88.7)	274.7 (108.6)		0.491	t-test
HCT, mean (SD)		42.7 (15.3)	42.3 (6.2)		0.757	t-test
MCV, mean (SD)		85.9 (9.4)	85.9 (13.6)		0.976	t-test
Presence of old lacunes, n, (%)	no	192, (80.7)	46, (19.3)		0.989	chi-square
	yes	233, (80.6)	56, (19.4)			
Presence of PVD, n, (%)	no	374, (80.8)	89, (19.2)	463	0.836	chi-square
	yes	51, (79.7)	13, (20.3)	64		
Use of rtPA, n, (%)	no	399, (82.1)	87, (17.9)	486	0.004	chi-square
	yes	26, (63.4)	15, (36.6)	41		
Presence of old microhaemorrhages, n (%)	no	395, (80.1)	98, (19.9)	493	0.247	chi-square
	yes	30, (88.2)	4, (11.8)	34		
Presence of microvascular changes, n (%)	no	158, (77.8)	45, (22.2)	203	0.196	chi-square
	yes	267, (82.4)	57, (17.6)	324		
Stroke classifications, n, (%)	TACS	18, (64.3)	10, (35.7)	28	<0.001	chi-square
	PACS	111, (64.5)	61, (35.6)	172		
	LACS	198, (94.7)	11, (5.30	209		
	POCS	92, (82.1)	20, (17.9)	112		
	undefined	6, (100)	0, (0)	6		
Size of artery, n, (%)	small	146, (94.8)	8, (5.2)	154	<0.001	chi-square
	large	279, (74.8)	94, (25.2)	373		

admission scan findings and biochemical parameters.

Abbreviations: NSAID = non-steroidal anti-inflammatory drugs, IHD = ischaemic heart disease, AMI = acute myocardial infarction, AF = atrial fibrillation, AKI = acute kidney injury, CKD = chronic kidney disease, HDL = high density lipoprotein, LDL = low density lipoprotein, Hb = haemoglobin, WBC = white blood cell counts, HCT = haematocrit, MCV = mean corpuscular volume, PVD = peripheral vascular disease, rtPA: recombinant tissue plasminogen activator, TACS = total anterior circulation syndrome, PACS = partial anterior circulation syndrome, LACS = lacunar syndrome, POCS = posterior circulation syndrome.

**Table 3:** Number of patients died in HT group during the follow-up period = 123.

	Non-HT, n = 425	HT, n = 102	p-value	test
No of patients died	123 (28.9%)	42 (41.2%)	0.017	Chi-square
Median survival, days (years)	4046 (11.1)	2757 (7.5)	0.010	Kaplan-Meier with log rank test

Number of patients died in No-HT group during the follow-up period =42.

**Figure 1:**
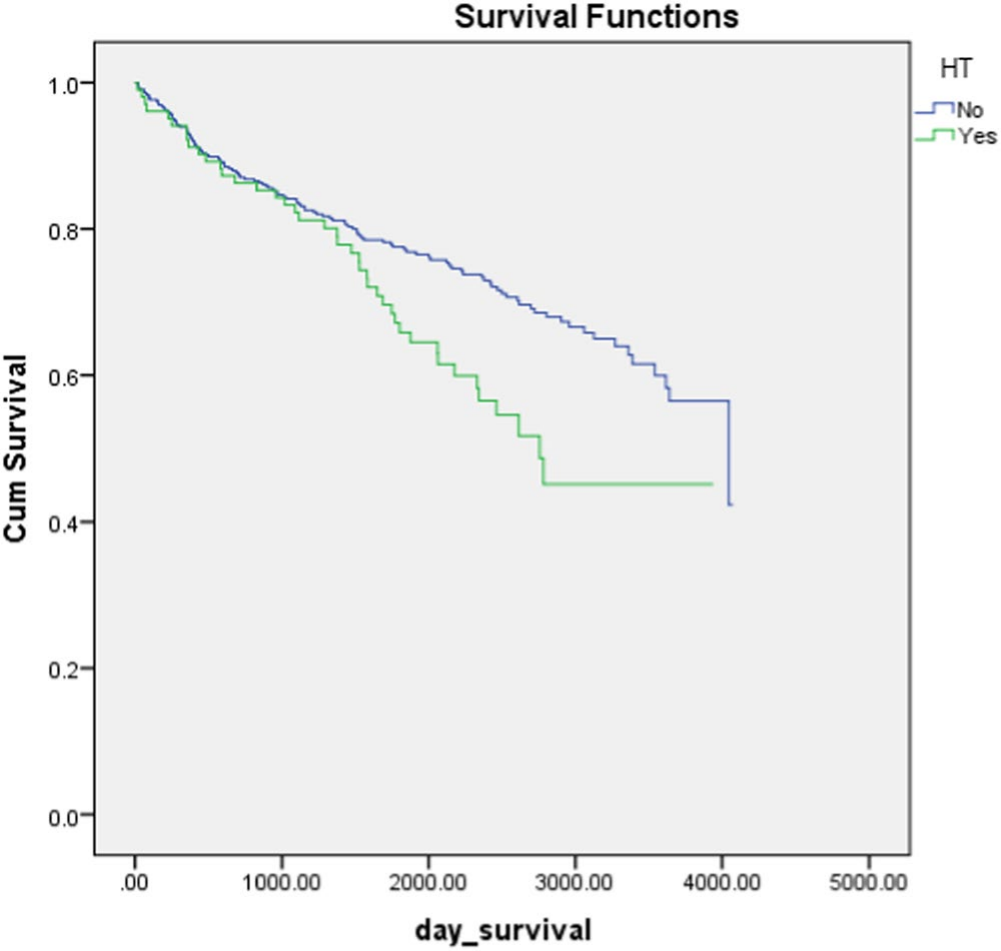
There was a significant difference in survival time between those who had HT and those who did not have HT (using Kaplan Meier survival analysis with log rank test, p = 0.010).

**Figure 2:**
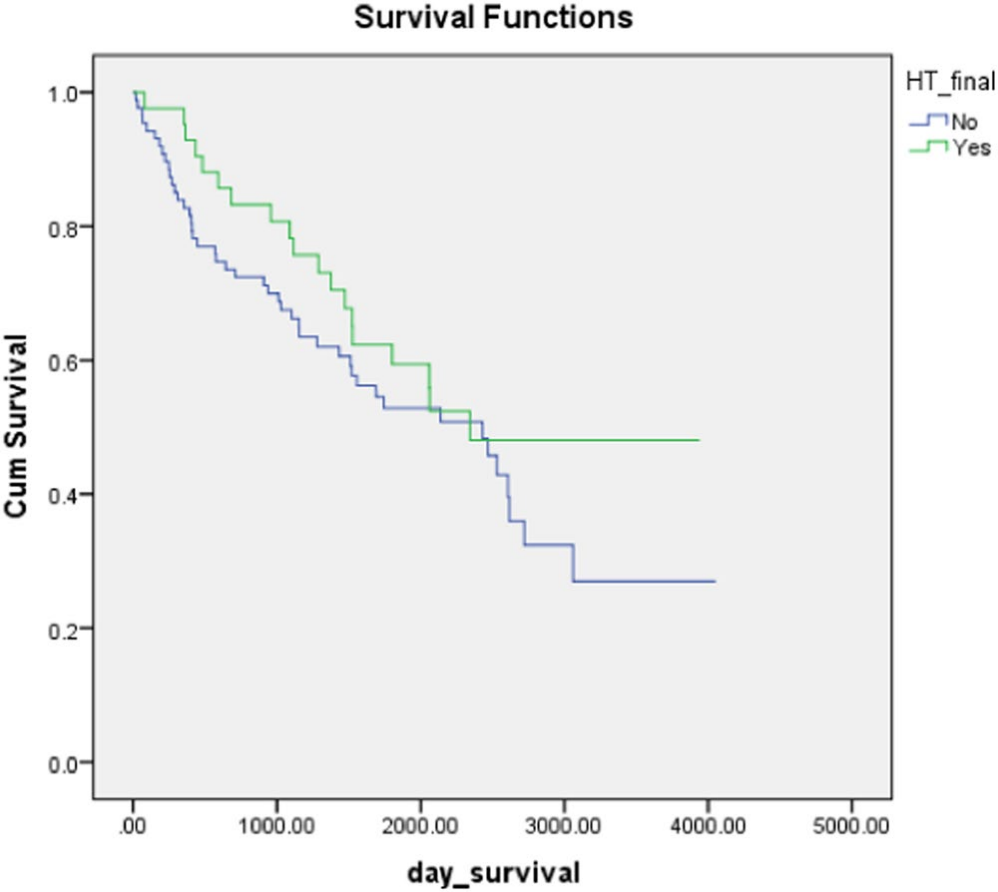
There was no longer statistically significant difference in survival time between HT and non-HT after adjusting for presence of comorbidities such as hypertension, diabetes and ischaemic heart disease (using Kaplan Meier survival analysis with log rank test, p = 0.291).

Mean survival was 7.5 years in the HT group as compared to 11.1 years in the stroke patients without HT (Table 3 and Fig. 1). However, after adjusting for comorbidities, the presence of HT was not significantly associated with survival (Fig. 2).

## Discussion

In the present study, 527 consecutive records of stroke patients who underwent rehabilitation were reviewed, and of these patients, 17.7% were diagnosed with HT. Studies from Singapore and south east Asian populations suggest that the frequency of haemorrhagic stroke is higher than in the Caucasian population^22^, however the overall frequency of ischaemic stroke remains high in Asian populations^23^. As compared to our data, in earlier studies by Hornig *et al*., HT was observed in 28 out of 65 patients (43%) over a four-week period^3^, and the frequency of HT was 29% in one neuropathological study^4^. The percentage of HT in studies of stroke patients varies from 6.4 to 43%^7,11,15,17,24–29^.

Thrombolytic treatment has been shown to increase the risk of haemorrhage and cause earlier HT^30^. Increased arterial stiffness has been found to be an independent predictor of HT in patients receiving thrombolysis^31^.

Data from studies of HT or SICH suggest an incidence of 6.4–30%, and up to 43% of combined HT and SICH cases^9,11,15,17,30^. In our study, thrombolysis with rtPA was performed in 39 (8%) patients, of which 16 (41%) developed HT. However, this may not be an accurate representation of the overall figures, as our study does not represent all patients. Some of the reasons for not undergoing thrombolysis following strokes in our study included late presentation to the emergency room, and patient and/or family refusal to receive thrombolysis due to the risk of associated bleeding.

We also found those with large artery infarctions to have a higher incidence of HT compared to those with small artery infarcts, which has also been described in previous studies^5,8,18^. Previous data suggest that HT is associated with thrombolysis treatment, cardioembolism, large artery infarcts, and severe neurological deficits. The authors of this study also concluded that stroke outcomes were in favour of patients developing HT and this was attributed to recanalisation and improved cerebral perfusion^32^.

We further analysed those patients who had cardioembolic risk factors, using the TOAST criteria. Our results suggest that there is a higher risk of HT in patients with known ischaemic heart disease and those with AF. A search of the literature on AF and stroke found evidence to show that patients with AF tended to have larger infarcts, as well as more frequent HT and hypoperfusion^33^, and an increased risk of PH^34^. In one study of 53 patients with cardioembolic stroke affecting MCA, HT occurred in 32%, and delayed recanalisation was associated with HT^35^.

Jan C *et al*. concluded that the risk of HT with novel oral anticoagulant (NOAC) use was similar in ischemic stroke patients who were treated with vitamin K antagonists to those who did not receive anticoagulant therapy^36^. In our study, we analysed patients who received vitamin k antagonists, NOACs, and dipyridamole for various underlying medical comorbidities. Our data suggest that vitamin k antagonists, is an independent risk factor for HT. However, we found no significant relationship between the use of NOACs, dipyridamole, or clopidogrel and HT. This may be due to the relatively small number of patients in this group. A significant number of patients presenting with stroke are elderly and may have associated underlying cardiovascular comorbidities. These people usually receive platelet inhibitors. The ECASS 2 study suggested that the extent of parenchymal hypoattenuation on cranial imaging after stroke is a risk factor for severe HT. Older patients^24,36^, and those who were on aspirin prior to stroke, are at higher risk of HT or parenchymal haemorrhage^37^ on rtPA.

In our study, we found that patients taking aspirin before being diagnosed with a stroke were more likely to experience HT. Old age was also associated with an increased risk of HT. Earlier studies failed to show an association between underlying microbleeds and HT after ischemic stroke^25^, while another study showed that more than three cerebral microbleeds were associated with PH and poorer outcomes^26^. The initial scan findings from our study were documented. Various findings were analysed and compared, including the presence of cerebral microbleeds and lacunes. We did not find a significant association between the presence of lacunes on imaging studies and HT.

Patients with stroke frequently have cardiovascular comorbidities, and take cholesterol-lowering agents. In a metanalysis, pre-treatment with statins has been shown to increase the risk of developing symptomatic HT in patients treated with thrombolysis^38^. We did not find any significant relationship between premorbid statin usage and the risk of HT. However, our analysis suggested that patients with lower total cholesterol and LDL values on admission had a higher incidence of HT. Other factors have been associated with HT, including high blood glucose, diabetes mellitus^18^, aspartate aminotransferase and bilirubin levels^27^, and body temperature^28^.

Our results suggest that full blood count at admission, including haemoglobin, white blood cells, platelets or altered liver function do not influence the risk of developing HT. We did not find any association between altered kidney function on admission and HT. Our data suggest that patients with HT have higher serum potassium levels.

A significant number of patients undergo inpatient rehabilitation following stroke. The authors of one study found that HT did not affect rehabilitation outcomes in stroke survivors^29^. We did not analyse functional outcomes in our cohort, due to incomplete data during the follow-up period; however, the survival data for our patients were analysed further. Early survival data from the ECASS I study suggested that PH patients have worse survival outcomes^39^. In our study, although there was a difference in survival outcomes between HT and other variants of stroke, after adjusting for comorbidities, HT did not influence survival.

Based on stroke severity, HT, and associated complications, some patients require neurosurgical interventions such as ICP management, EVD insertion, and craniotomy or craniectomy. The survival outcomes of patients undergoing neurosurgical intervention was not significantly different.

Minocycline has been shown to inhibit matrix metalloproteinases and reduce HT in rodents following rtPA, and the role of minocycline has been studied in the WAIMATSS multicenter trial^40^.

Despite the selection bias, the findings of our study including the incidence and comorbidities in relation to HT flowing ischaemic strokes are in line with prospective and retrospective studies undertaken previously.

Locally available data suggest that thrombolysis rates in acute ischemic stroke are low. There are multifarious reasons, however, the risk of haemorrhage seems to be the main factor.

Although some of the studies conclude that HT suggests recanalisation of the arteries following ischaemic stroke however, these patients need careful monitoring as some of them may need medical or neurosurgical intervention.

Furthermore, initiation of the treatment with antiplatelets and anticoagulants is dependent on resolution of HT. Consequently, we believe that this study can lead to the timely screening of patients for thrombolysis and provide guidance to at-risk patients for closer monitoring for HT regardless of Thrombolysis.

The strengths of our study include the fact that it was carried out in a government-restructured hospital, and thus the study sample is representative of a wide range of ages, genders, and ethnicities. Previous studies on patients with ischaemic and haemorrhagic stroke from the local population are available for comparison. To the best of our knowledge, this study is the first to review HT in acute ischaemic stroke, and its relationship with comorbidities, particularly in patients with and without thrombolysis.

The limitations of this study include retrospective nature of the data collection. There may also be a selection bias, in that we only reviewed patients admitted to a neurological rehabilitation centre. Some patients with minimal neurological deficits were discharged with no follow-up cranial imaging, and those who passed away following admission are not included in our study, hence HT in this group may be underrepresented. Finally, functional outcomes were not available for analysis.

## Conclusion

Our study confirms that there is an increased risk of HT with advancing age, large artery infarcts, treatment with rtPA, and underlying cardiovascular risk factors, especially AF. Premorbid antiplatelets and vitamin k antagonists, also show a strong correlation with HT. Survival rates were not affected by the presence of HT. The role of low total cholesterol and LDL in HT requires further study.

We are now analysing functional outcomes in our patients, including those patients with intracerebral haemorrhage.

## Data Availability

We would seek Singhealth IRB approval for data submission.
